# Correlation analysis of diabetes based on Copula

**DOI:** 10.3389/fendo.2024.1291895

**Published:** 2024-02-14

**Authors:** Chang Liu, Hu Yang, Junjie Yang, Hongqing Wang

**Affiliations:** ^1^ College of Science, Beijing Forestry University, Beijing, China; ^2^ State Key Laboratory of Vegetation and Environmental Change, Institute of Botany, Chinese Academy of Sciences, Beijing, China

**Keywords:** fasting blood glucose, glycosylated hemoglobin, triglyceride, high-density lipoprotein cholesterol, nonlinear correlation

## Abstract

**Introduction:**

The ratio of Triglyceride (TG) to high-density lipoprotein cholesterol (HDL-C) is a crucial indicator for diabetes diagnosis.

**Methods:**

This study utilizes the Copula function to model and fit the non-linear correlation among fasting blood glucose (Glu), glycosylated hemoglobin (HbA1C), and TG/HDL-C in patients with diabetes. The Copula function chosen for this study includes the two-dimensional Archimedes and Elliptical distribution family, as well as the multidimensional Vine Copula function, for fitting the data. The evaluation of the fitting effect is performed using the mean absolute error (MAE) and mean square error (MSE).

**Results:**

The results indicate that the Clayton Copula exhibits the highest effectiveness in fitting the pairwise relationship between Glu and TG/HDL-C, as well as HbA1C and TG/HDL-C, displaying the smallest fitting error. Additionally, the Vine Copula function produces a satisfactory fit for the relationship among all three indicators. Compared to linear analysis methods, the Copula function more accurately depicts the correlation among these three types of indicators.

**Discussion:**

Moreover, our findings indicate a stronger correlation in the lower tail between Glu and HbA1C, as well as TG/HDL-C, suggesting that the Copula function provides greater accuracy and applicability in depicting the relationship among these indicators. As a result, it can offer a more precise auxiliary diagnosis and serve as a valuable reference in clinical judgment.

## 1 Introduction

Diabetes is a metabolic disease characterized by hyperglycemia resulting from defective insulin secretion or damage to the pancreatic islets (1). A recent report indicates that the global population with diabetes reached approximately 537 million in 2021, and this number is continuously increasing at a rapid pace (2, 3). Due to the complex nature of the disease, there is currently no medical cure for diabetes. Instead, treatment focuses on regulating blood glucose levels through various methods of control and management. Diabetes is prominently listed among the top 10 causes of death globally, according to the World Health Organization’s 2019 report. Given the prevalence and treatment challenges associated with diabetes, it is crucial to explore its underlying causes and indicators, detect early signs, and intervene to mitigate its progression.

Chronically elevated blood glucose level in diabetic patients often leads to abnormal lipid metabolism, typically marked by increased Triglyceride (TG) concentration and decreased high-density lipoprotein cholesterol (HDL-C) concentration (2, 4). He et al. were the first to propose that TG/HDL-C is a more effective predictor of diabetes compared to individual TG or HDL-C levels, representing an independent risk factor for diabetes (5). Researchers typically employ one-way or multi-factorial analysis of variance, Pearson correlation analysis, linear analysis methods, and Cox proportional risk models to test the validity of their hypotheses and determine significant differences between factors, aiming to establish independent predictors of a given condition (4, 6–9). For example, in a study involving a test-control group, patient information such as age, gender, and Body Mass Index (BMI) is recorded. Logistic analysis modeling is then employed to analyze fasting blood glucose (Glu), 2-hour postprandial blood glucose (2hPG), glycosylated hemoglobin (HbA1C), TG, total cholesterol (TC), and HDL-C concentrations. Multifactorial analysis reveals a significant positive correlation between TG/HDL-C and Glu as well as HbA1C, highlighting the former as an important predictor of diabetes (2, 5, 10). Furthermore, some researchers have discovered nonlinear relationships among different indicators that cannot be adequately explored using linear analysis methods alone. For example, Chen noted the complex relationship between TG/HDL-C, Glu, and HbA1C and employed a generalized additive model to investigate this relationship further; however, the nonlinear correlation has yet to be thoroughly explored (2).

Copula function, a tool commonly used in probability theory and statistics, is primarily utilized for describing and modeling the joint probability distribution function of multi-dimensional random variables. It combines each marginal distribution with a function called Copula to represent the joint distribution of multi-dimensional random variables, which has the characteristics of flexible structure, simple solution, wide applicability, etc. It can fit the joint distribution of a variety of different distribution variables. At present, it is widely used in nonlinear relationships in finance, wind power, water conservancy, and other fields, but is still poorly used in the medical field (11–13). Espasandín-Domínguez et al. proposed the use of a generalized nonlinear additive model and Copula function to establish the dual response variable of glycosylated hemoglobin and fructosamine to investigate their relationship with blood glucose, providing valuable clinical insights (14). Robinson Dettoni et al. employed a semi-parametric recursive copula to model the relationship between obesity and related conditions such as hypertension, hyperlipidemia, and diabetes using the theory of health production. The results indicate that reducing obesity rates could help decrease morbidity and mortality rates associated with these diseases (15). In current clinical trials, it has been found that TG/HDL-C has a certain correlation with various indicators in patients, and the ratio can reflect the changes in blood glucose concentration to a certain extent (5, 9, 16). However, general linear methods struggle to accurately represent the complex nonlinear relationship among TG/HDL-C, Glu, and HbA1C. Consequently, there have been no studies exploring the nonlinear correlation between these three variables utilizing Copula functions. Thus, this paper aims to further investigate the nonlinear relationship between TG/HDL-C, Glu, and HbA1C in patients using Copula functions. We hypothesize that when TG/HDL-C levels are elevated, the correlations between these three variables strengthen, offering new perspectives for the auxiliary diagnosis of diabetes.

## 2 Materials and methods

### 2.1 Description of data

The data used in this study is obtained from the National Data Center for Population Health Sciences Data warehouse PHDA 2022, (https://www.ncmi.cn//phda/dataDetails.do?id=CSTR: *A*0006.11*.A*0005.201905.000282), a diabetes complication early warning dataset uploaded by the General Hospital of the Chinese People’s Liberation Army. This dataset includes de-identified information for three thousand patients, encompassing personal details such as age, gender, ethnicity, and marital status. Additionally, it comprises measurements of Glu, HbA1C, TC, TG, HDL-C, and low-density lipoprotein cholesterol (LDL-C), all of which were appropriately processed and standardized.

### 2.2 Application of copula function in this paper

Sklar’s definition of the Copula function highlights its critical role in one-dimensional and multidimensional joint distributions (17). In short, a joint distribution function *H* with marginal distribution functions *F* and *G* must have a unique Copula function such that:


H(x,y) =C(F(x,y),G(x,y)).

Different Copula functions serve different purposes, depending on the characteristics of the variables’ distribution. For instance, the Clayton Copula function’s asymmetric tail characteristics capture the asymmetric tail correlation between random variables and are more responsive to distribution changes in the lower tail. If the Clayton Copula function portrays the correlation structure between two variables, the lower tail of the distribution has a strong correlation. For joint distributions with three or more dimensions, the Vine Copula function describes many correlations between variables, and the number of correlations it describes increases as the dimension of the joint distribution grows, making its advantages more pronounced.

This study primarily uses Copula functions from the two-dimensional Archimedes and Elliptical families for modeling the relationship between TG/HDL-C and Glu and between TG/HDL-C and HbA1C. To examine the nonlinear relationship among TG/HDL-C, Glu, and HbA1C, we utilize the Vine Copula function for multidimensional data.

If the Copula function effectively models the nonlinear correlation between TG/HDL-C, Glu, and HbA1C, it suggests a robust nonlinear correlation among the three. Thus, TG/HDL-C can serve as an auxiliary diagnostic indicator of diabetes, aiding in clinical prevention and diagnosis of diabetes.

### 2.3 Box-Cox transform

Box-Cox transform is a widely used method for data transformation, primarily employed to convert non-normally distributed datasets into approximately normally distributed ones(18). By adjusting the power parameters, the data is transformed as follows:


$$y\left(\text{λ}\right)=\{\begin{array}{l} \frac{{\left(y+\varepsilon \right)}^{\text{λ}}+1}{\text{λ}},\text{λ}\neq 0\\ \log\;\left(y\right),\text{λ}=0 \end{array},$$

where *y* denotes the original data, *λ* is the idempotent parameter and *ε* is a constant to ensure that the data are all positive. If *λ* = 0, it is a logarithmic transformation. In this paper, we utilize the method of minimizing the difference between skewness and kurtosis to determine the optimal *λ* value.

The Box-Cox transformation enhances the likelihood of the data conforming to normal distribution characteristics. Additionally, it aids in reducing unobservable errors in the data, thereby mitigating potential errors arising from manually recorded patient information. This process facilitates subsequent analysis in this paper.

## 3 Results

**Figure 1:**
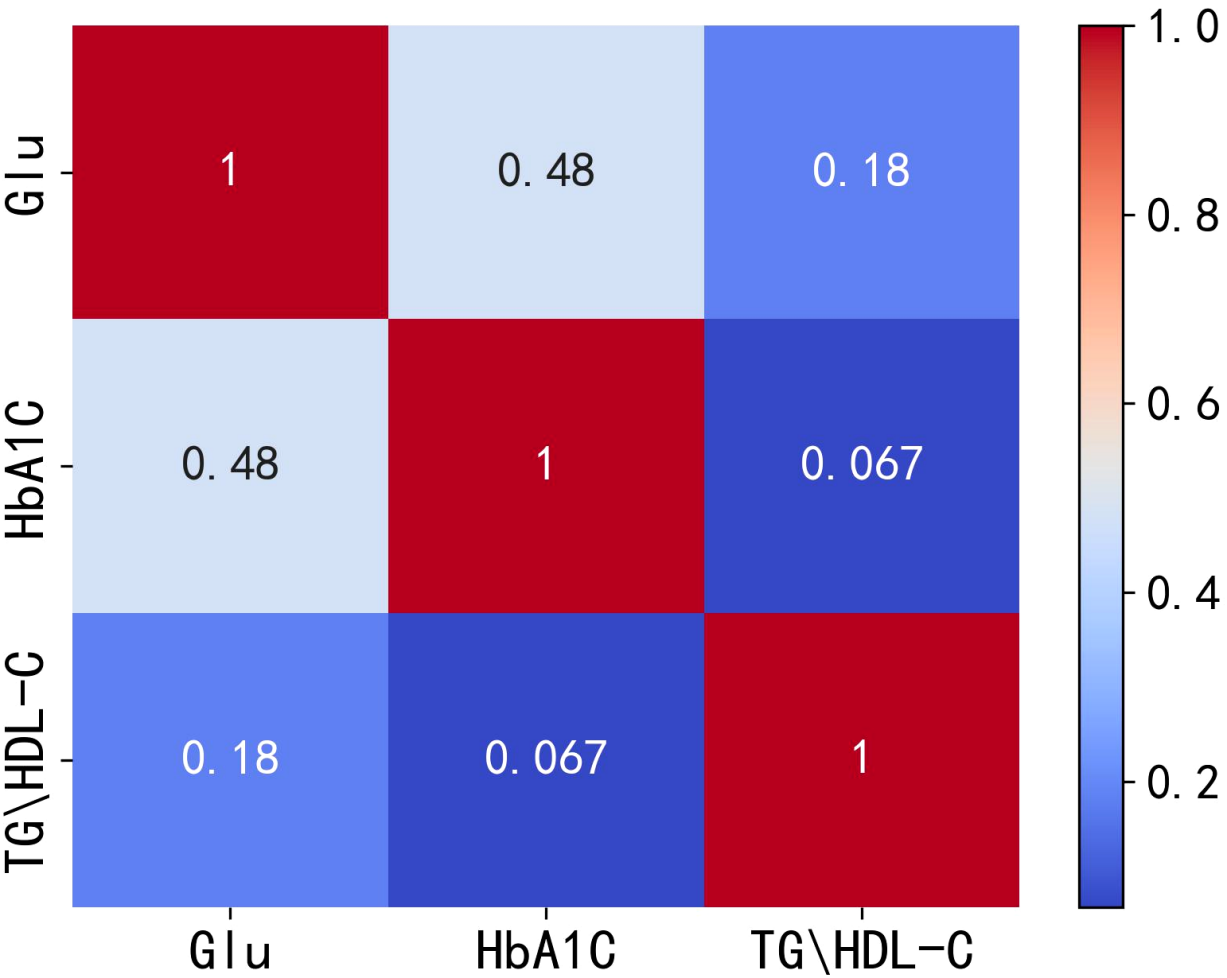
Heat map of correlation between Glu, HbA1C and TG/HDL-C. Glu, fasting blood glucose; HbA1C, glycosylated hemoglobin; TG/HDL-C, the ratio of Triglyceride(TG) to high-density lipoprotein cholesterol(HDL-C).

According to international standards, Glu and HbA1C are currently used as important indicators for the diagnosis of diabetes mellitus (19–21). Therefore, in this paper, Glu and HbA1C are considered as the dependent variables, and TG/HDL-C is analyzed as the independent variable (2, 3, 10). After addressing missing values by either removing or filling them, and eliminating outliers using the 3*σ* principle, a total of 2,901 valid data points are obtained. The present study then extracts and calculates the patients’ Glu, HbA1C, and TG/HDL-C levels. Given that the internal indicators of patient data generally exhibit nonlinear relationships, the Spearman correlation coefficient is more representative of such relationships. Consequently, this paper conducts a Spearman correlation analysis among these three variables, and the results are depicted in **Figure 1**.

The correlation analysis plots indicate a positive correlation between TG/HDL-C and both Glu and HbA1C, with correlation coefficients of 0.18 and 0.067, respectively. However, these correlation coefficients are relatively low, suggesting that the positive correlation between TG/HDL-C and Glu or HbA1C is not very strong. Therefore, it can be speculated that the positive correlation between TG/HDL-C and both Glu and HbA1C is not completely independent of each other. To test the hypothesis of independence, the following hypotheses are considered:

the original hypothesis *H*_0_: TG/HDL-C is independent of both Glu and HbA1C;
the alternative hypothesis *H*_1_: TG/HDL-C is not independent of either Glu or HbA1C.

The results reveal a P value of 7.6362*e-13(***), which leads to the rejection of the original hypothesis and acceptance of the alternative hypothesis. This indicates that TG/HDL-C is indeed not independent of both Glu and HbA1C.

Considering the presence of correlation among the three variables, albeit with low correlation coefficients, and the lack of independence between TG/HDL-C and Glu or HbA1C, this study suggests the existence of a nonlinear relationship. To further analyze the correlation among these indicators in diabetic patients, the Copula function is introduced, which is known for its effectiveness in handling nonlinear relationships (17).

### 3.1 Marginal distribution of the fitted variables

**Figure 2:**
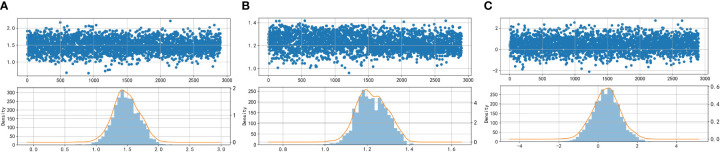
Scatter plots and histograms of density distributions of Glu **(A)**, HbA1C **(B)**, and TG/HDL-C **(C)**. Glu, fasting blood glucose; HbA1C, glycosylated hemoglobin; TG/HDL-C, the ratio of Triglyceride(TG) to high-density lipoprotein cholesterol(HDL-C). When the curves are approximately normally distributed, the data can be considered to be approximately fitted with a normal distribution.

**Figure 3:**
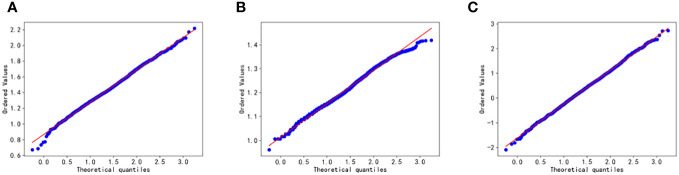
Q-Q plot test for Glu **(A)**, HbA1C **(B)**, and TG/HDL-C **(C)**. Glu, fasting blood glucose; HbA1C, glycosylated hemoglobin; TG/HDL-C, the ratio of Triglyceride(TG) to high-density lipoprotein cholesterol(HDL-C). Data are considered to pass the Q-Q plot normality test when the data points fall approximately on a straight line.

In the paper, the Box-Cox transform is applied to normalize the data. Following the transformation, scatter plots, density distribution histograms, and Q-Q plots are utilized to assess the distribution of the variables. The closer the density distribution histogram resembles a normal curve and the more the data points in the Q-Q plots align with a straight line, the more indicative it is of the variables conforming to a normal distribution. **Figures 2** and **3** present scatter plots, density distribution histograms, and Q-Q plots for TG/HDL-C, Glu, and HbA1C. These visualizations provide a rough evaluation of the distribution characteristics of the variables after the Box-Cox transform.

Based on **Figures 2** and **3**, it can be preliminarily observed that the density distribution histogram of Glu, HbA1C, and TG/HDL-C approximately conforms to a normal distribution curve, and the data points in the Q-Q plots mostly align with a straight line. This suggests that these variables may follow a normal distribution. To confirm the normality of these variables, a normality test is performed. **Table 1** presents the results of the normality test for Glu, HbA1C, and TG/HDL-C.

**Table 1:** Tests for normal distribution of Glu, HbA1C and TG/HDL-C variables.

Variable	K-S test		Normality test	
	pass or fail	p-value	pass or fail	p-value
Glu	pass	0.1967	pass	0.7598
HbA1C	pass	0.2771	pass	0.9008
TG/HDL-C	pass	0.8942	pass	0.8205

Glu, fasting blood glucose; HbA1C, glycosylated hemoglobin; TG/HDL-C, the ratio of Triglyceride(TG) to high-density lipoprotein cholesterol(HDL-C).

**Table 1** indicates that the p-values from the K-S test and normality test for Glu, HbA1C, and TG/HDL-C variables are greater than 0.05, which suggests that there is no significant evidence to reject the hypothesis of conformity to a normal distribution for these variables. Therefore, based on the test results, it can be concluded that all three variables conform to a normal distribution.

### 3.2 Fitting the edge distribution of the variables

According to the previous section, the study establishes that all three variables can be fitted with normal distribution, so in this section, this paper is going to select the appropriate copula function for fitting the joint distribution function of TG/HDL-C and Glu, as well as TG/HDL-C and HbA1C, and use the appropriate indexes to assess the goodness of fit.

#### 3.2.1 Copula fitting of TG/HDL-C variables to Glu variables

**Figure 4:**
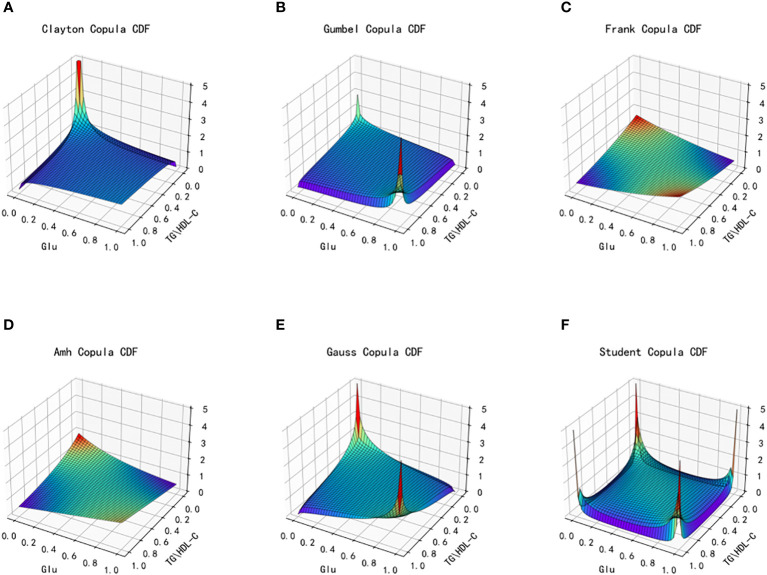
Joint distribution function images of six copulas fitting for TG/HDL-C and Glu. **(A)** Clayton Copula, **(B)** Gumbel Copula, **(C)** Frank Copula, **(D)** Ali–Mikhail–Haq Copula, **(E)** Gauss Copula, **(F)** student Copula. TG/HDL-C, the ratio of Triglyceride(TG) to high-density lipoprotein cholesterol(HDL-C); Glu, fasting blood glucose.

**Figure 5:**
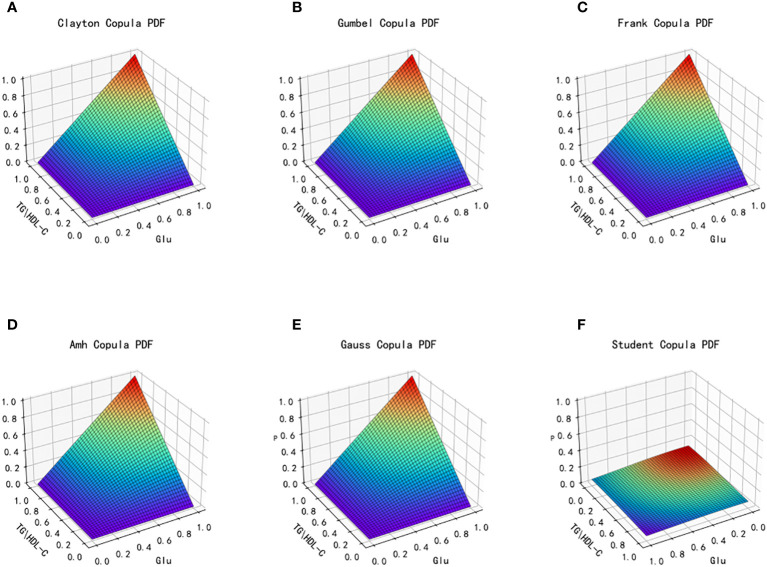
Probability distribution function images of six copulas fitting for TG/HDL-C and Glu. **(A)** Clayton Copula, **(B)** Gumbel Copula, **(C)** Frank Copula, **(D)** Ali–Mikhail–Haq Copula, **(E)** Gauss Copula, **(F)** student Copula. TG/HDL-C, the ratio of Triglyceride(TG) to high-density lipoprotein cholesterol(HDL-C); Glu, fasting blood glucose.

Based on the different characteristics of the two types of index data, TG/HDL-C and Glu, in the dataset, a total of six copulas are selected for fitting, including Clayton Copula, Gumbel Copula, Frank Copula, Ali–Mikhail–Haq Copula in Archimedes Copula, Gauss Copula and Student(t) Copula in Elliptical Copulas. Copula function’s density distribution and joint distribution images are produced. We use MAE and MSE to evaluate the fitting effect of each type of copula function and the smaller these indicators, the better the fit. The images of the joint distribution function and the probability distribution function obtained after fitting using the six copula functions mentioned above are depicted in **Figures 4** and **5**, respectively.

**Table 2** presents the goodness of fit results for Clayton Copula, Gumbel Copula, Frank Copula, Ali–Mikhail–Haq Copula, Gauss Copula, and t Copula. The table includes the MAE and MSE values.

**Table 2:** Effect of copula function fitting for TG/HDL-C and Glu.

Types of copula functions	Copula function name	MAE	MSE
Archimedes Copula	Clayton Copula	0.6613	0.5880
	Gumbel Copula	0.7915	0.8308
	Frank Copula	0.7827	0.8152
	Ali–Mikhail–Haq Copula	0.7881	0.8188
Elliptical Copula	Gauss Copula	0.7880	0.8273
	Student(t) Copula	0.7819	0.8109

TG/HDL-C, the ratio of Triglyceride(TG) to high-density lipoprotein cholesterol(HDL-C); Glu, fasting blood glucose.

From the fitted images and the comparison of simulation fitting errors among the six copula functions, it is evident that copula functions are effective in fitting the correlation between TG/HDL-C and Glu. Among these, Clayton Copula produces the smallest error in both categories and exhibits the best-fitting effect for TG/HDL-C and Glu.

#### 3.2.2 Copula fitting of TG/HDL-C variables to HbA1C variables

**Figure 6:**
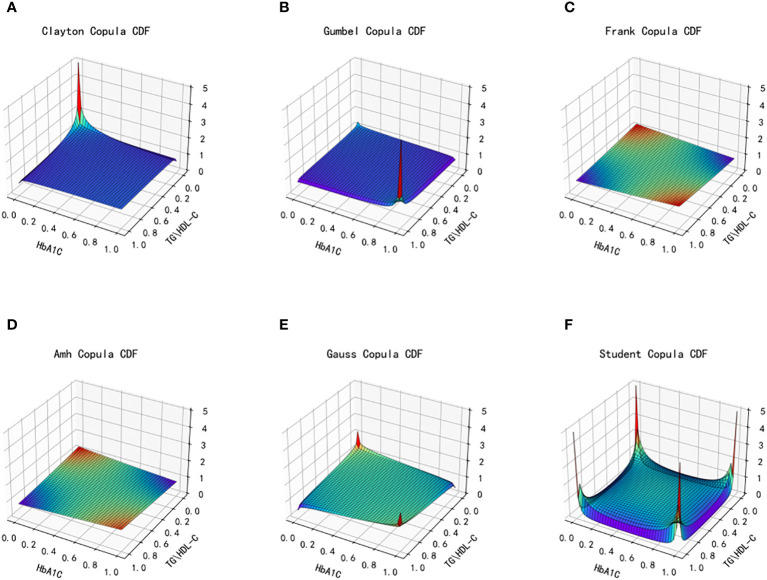
Joint distribution function images of six copulas fitting for TG/HDL-C and HbA1C. **(A)** Clayton Copula, **(B)** Gumbel Copula, **(C)** Frank Copula, **(D)** Ali–Mikhail–Haq Copula, **(E)** Gauss Copula, **(F)** student Copula. TG/HDL-C, the ratio of Triglyceride(TG) to high-density lipoprotein cholesterol(HDL-C); HbA1C, glycosylated hemoglobin.

**Figure 7:**
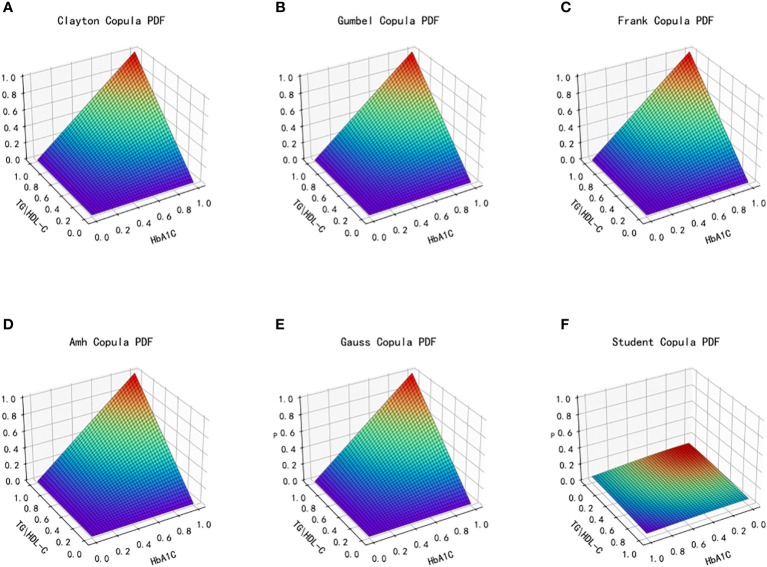
Probability distribution function images of six copulas fitting for TG/HDL-C and HbA1C. **(A)** Clayton Copula, **(B)** Gumbel Copula, **(C)** Frank Copula, **(D)** Ali–Mikhail–Haq Copula, **(E)** Gauss Copula, **(F)** student Copula. TG/HDL-C, the ratio of Triglyceride(TG) to high-density lipoprotein cholesterol(HDL-C); HbA1C, glycosylated hemoglobin.

The dataset’s distinct characteristics of the two types of data, TG/HDL-C and HbA1C, lead to the selection of six copulas for fitting: Clayton Copula, Gumbel Copula, Frank Copula, Ali–Mikhail–Haq Copula in Archimedes Copula, Gauss Copula, and t Copula in Elliptical Copulas. Subsequently, the density distribution of each copula function is generated after fitting, resulting in the production of the density distribution function and joint distribution function images. Evaluation of the fitting effect for each type of copula function is conducted using MAE and MSE, with smaller values indicating better fitting. The graphical representation of the fit and effect of the six copula functions is depicted in **Figures 6** and **7**, showing the images of the joint distribution function and the probability distribution function obtained after fitting using the selected copula functions.

**Table 3** presents the goodness of fit results for Clayton Copula, Gumbel Copula, Frank Copula, Ali–Mikhail–Haq Copula, Gauss Copula, and t Copula. The table includes the MAE and MSE values.

**Table 3:** Effect of copula function fitting for TG/HDL-C and HbA1C.

Types of copula functions	Copula function name	MAE	MSE
Gumbel Copula	Clayton Copula	0.4190	0.3794
	Archimedes Copula	0.5932	0.4975
	Frank Copula	0.5916	0.4929
	Ali–Mikhail–Haq Copula	0.4962	0.4064
Elliptical Copula	Gauss Copula	0.5954	0.4941
	t Copula	0.5890	0.4857

TG/HDL-C, the ratio of Triglyceride(TG) to high-density lipoprotein cholesterol(HDL-C); HbA1C, glycosylated hemoglobin.

After fitting the images of the six copula functions and comparing the simulated fitting errors for the different copula functions, it can be concluded that copula functions effectively fit the correlation between TG/HDL-C and HbA1C. Clayton Copula produced the smallest error in both categories and exhibited the best fit for TG/HDL-C and HbA1C.

#### 3.2.3 Fitting between TG/HDL-C, Glu and HbA1C

**Figure 8:**
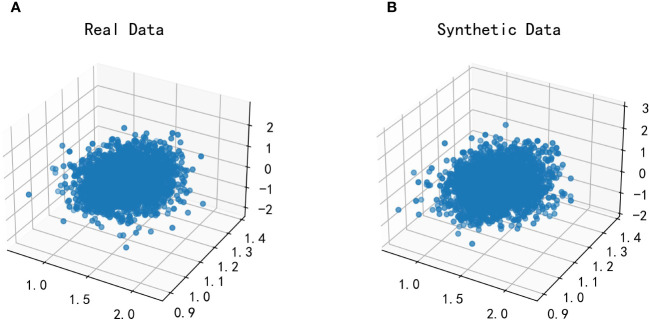
Plot of the effect of fitting the Vine Copula function for TG/HDL-C, Glu and HbA1C. Glu, fasting blood glucose; HbA1C, glycosylated hemoglobin; TG/HDL-C, the ratio of Triglyceride(TG) to high-density lipoprotein cholesterol(HDL-C). **(A)** is the scatter plot of the real dataset in this paper; **(B)** is the scatter plot of the randomized data generated after fitting by the Vine Copula function.

For modeling the function with both Glu and HbA1C variables as dependent variables and TG/HDL-C as an independent variable, a multidimensional Copula function can be utilized to achieve a good fit. In this paper, Vine Copula is selected for fitting based on data characteristics. However, due to the high dimensionality of the data, the joint distribution function and probability distribution function of the data could not be displayed graphically. To overcome this challenge, we employ the fitted three-dimensional Vine Copula to generate random numbers, which are then plotted against the dataset of this study for comparison. **Figure 8** depicts the scatter plot of this dataset with the generated random data from the fitted Copula function.

By comparing the two scatter plots, it can be observed that there is no significant difference between the left and right plots. This finding suggests that Vine Copula provides a better fit for Glu, HbA1C, and TG/HDL-C variables.

### 3.3 Analysis of results

The six selected copula functions fit TG/HDL-C to Glu, TG/HDL-C to HbA1C, and the joint distribution of the three relatively well, with both types of errors (MAE and MSE) remaining within acceptable limits. Among them, the best fit is the Clayton Copula, which exhibits the lowest errors among the six copula functions. The parameters in the Clayton Copula function obtained by modeling TG/HDL-C and Glu, and TG/HDL-C and HbA1C are as follows, respectively:


$$\begin{array}{l} \theta_{Glu}=\;0.2146;\\ \theta_{HbA1C}=\;0.0525. \end{array}$$

This results in two Clayton Copula modeling function formulas, one obtained by modeling TG/HDL-C and Glu, and the other obtained by modeling TG/HDL-C and HbA1C:


$$C_{1}\left(u_{Glu},u_{TG/HDL-C};\theta_{Glu}\right)=\max\;\left\{{\left(u_{Glu}^{-\theta_{Glu}}+u_{TG/HDL-C}^{-\theta_{Glu}}-1\right)}^{-\frac{1}{\theta_{Glu}}},0\right\};$$

$$C_{2}\left(u_{HbA1C},u_{TG/HDL-C};\theta_{HbA1C}\right)=\max\;\left\{{\left(u_{HbA1C}^{-\theta_{HbA1C}}+u_{TG/HDL-C}^{-\theta_{HbA1C}}-1\right)}^{-\frac{1}{\theta_{HbA1C}}},0\right\}.$$

In Section 2, we discussed that the Clayton Copula is particularly sensitive to changes in the lower tail of variable distribution, and can more accurately reflect changes in the lower tail between variables (22). In our study, the lower tail portion of the variables is evident by an increase in TG/HDL-C accompanied by an increase in Glu or HbA1C. As the values of TG/HDL-C, Glu, or HbA1C increase, the nonlinear correlation between the three becomes stronger, indicating a significant correlation. Therefore, it can be concluded that TG/HDL-C is an important predictor of diabetes.

### 3.4 The linear analysis method fitting

Commonly used statistical methods in the medical field include one-way and multivariate analysis of variance, as well as simulations utilizing logistic analysis methods to obtain variable predictors and variability in trial data (23, 24). However, these methods fail to fully consider the complex nonlinear correlations between variables, often leading to overlooked factors or disregarded complex relationships, resulting in a poor fit and imprecise responses to variable changes. For the characteristics of this dataset, we opt for the linear analysis method to construct a simulation fit and compare it with the Copula functions used in this study.

TG/HDL-C is modeled as the independent variable, while Glu and HbA1C are modeled as dependent variables, respectively. Subsequently, we obtain the following linear analysis model equations:


$$\begin{array}{l} {\hat{y}}_{Glu}=\;0.2355x+\;7.9132;\\ {\hat{y}}_{HbA1C}=\;0.0699x+\;7.6585. \end{array}$$

For the established models, the same two metrics, MAE and MSE, are used to assess the fit of the models, and **Table 4** shows a table of the effect of the linear analysis methods fitting TG/HDL-C to Glu and TG/HDL-C to HbA1C.

**Table 4:** Table of the linear analysis method fitting effects of TG/HDL-C with Glu and TG/HDL-C with HbA1C.

Class of equations	MAE	MSE
TG/HDL-C to Glu	2.7700	15.5800
TG/HDL-C to HbA1C	1.4553	7.4578

TG/HDL-C, the ratio of Triglyceride(TG) to high-density lipoprotein cholesterol(HDL-C); Glu, fasting blood glucose; HbA1C, glycosylated hemoglobin.

By examining the fitting errors of the linear analysis method, it is determined that the model’s fitting errors for TG/HDL-C to Glu and TG/HDL-C to HbA1C both exceed 1, indicating substantial errors and an acceptable fit. Conversely, the six copula functions discussed in the previous section exhibit fitting errors all below 1. Among them, the best-fitting Clayton Copula function yields errors of 0.6613 (MAE), 0.5880 (MSE) for TG/HDL-C to Glu, and 0.4190 (MAE), 0.3794 (MSE) for TG/HDL-C to HbA1C. These results unequivocally demonstrate that the fitting effect of Copula functions surpasses that of the linear analysis method commonly employed in medical research.

### 3.5 Construct the linear analysis models and Copula function methods

**Table 5** presents the simulation error tables constructed for the Copula function and the linear analysis method, demonstrating their application to TG/HDL-C to Glu and TG/HDL-C to HbA1C in this paper.

**Table 5:** Table of fitting effects of seven types of functions.

Function type	TG/HDL-C to Glu		TG/HDL-C to HbA1C	
	MAE	MSE	MAE	MSE
Clayton Copula	0.6613	0.5880	0.4190	0.3794
Gumbel Copula	0.7915	0.8308	0.5932	0.4975
Frank Copula	0.7827	0.8152	0.5916	0.4929
Ali–Mikhail–Haq Copula	0.7881	0.8188	0.4962	0.4064
Gauss Copula	0.7880	0.8273	0.5954	0.4941
t Copula	0.7819	0.8109	0.5890	0.4857
the linear analysis method	2.7700	15.5800	1.4553	7.4578

TG/HDL-C: the ratio of Triglyceride(TG) to high-density lipoprotein cholesterol(HDL-C); Glu:fasting blood glucose; HbA1C:glycosylated hemoglobin.

The error data obtained from fitting the linear analysis method in the aforementioned table indicates an inadequate correlation between TG/HDL-C and Glu, as well as TG/HDL-C and HbA1C. This suggests that the linear analysis method fails to adequately capture the intricate relationship between these indicators. A comparison with the previous error table for the copula function’s fitting reveals that the Copula function is more accurate in depicting the nonlinear correlations among Glu, HbA1C, and TG/HDL-C indicators in diabetic patients. This is attributed to the Copula function’s capability to capture intricate nonlinear relationships and tail correlations. In this study, the Copula function effectively demonstrates its capacity to identify intricate nonlinear relationships among TG/HDL-C, Glu, and HbA1C indicators. Particularly in the lower tail section, when the TG to HDL-C ratio is higher, the Copula function accurately represents the changes in Glu and HbA1C indicators, which exhibit stronger nonlinear correlations with it. Consequently, the Copula function proves to be more precise in the analysis of nonlinear correlations among TG/HDL-C, Glu, and HbA1C indicators. Consequently, the implementation of the Copula function in exploring the patient’s internal index data yields more significant results. It enables the analysis and identification of nonlinear relationships in the patient’s data through a closer fitting effect, consequently offering more precise predictions and diagnostic references in clinical judgment.

## 4 Discussion

This study utilizes Copula functions to investigate the nonlinear relationships among three key indicators in diabetic patients: Glu, HbA1C, and TG/HDL-C. A comprehensive data analysis reveals that the Clayton Copula demonstrates particular sensitivity to the lower tail dependence of these indicators, effectively reflecting their variations. Remarkably, an elevated TG/HDL-C exhibits a significant correlation with both Glu and HbA1C, highlighting the predictive significance of TG/HDL-C for diabetes. In comparison to traditional linear analysis methods, Copula functions excel at capturing the intricate nonlinear interrelationships among these indicators, particularly about extreme values.

From a biological standpoint, the relationships among these indicators signify prevalent biochemical alterations in metabolic syndrome, encompassing insulin resistance, dysregulated blood sugar control, and abnormalities in lipid metabolism. Diabetic patients with a high TG to HDL-C ratio exhibit a close association with elevated levels of Glu and HbA1C, unveiling intricate physiological interactions among these markers (2, 5, 6, 9, 10, 25, 26–28). This imparts vital information for clinicians, fostering a more comprehensive comprehension of the mechanisms underpinning diabetes and its complications, and enabling more precise diagnoses and personalized treatment plans for patients (29–32).

One of the most significant findings in this study is that employing Copula functions has facilitated a comprehensive comprehension of the intricate interactions among these pivotal indicators in diabetic patients. Nevertheless, our research possesses limitations, encompassing a limited scope of data and inadequate consideration of other pertinent biochemical markers. Subsequent studies ought to augment the sample size, incorporate a greater number of relevant indicators, and employ longitudinal data to further corroborate our findings. Furthermore, implementing these findings in clinical practice for wider validation and assessment also signifies a crucial avenue for future research.

In summary, by conducting a comprehensive analysis of the Glu, HbA1C, and TG/HDL-C indicators in diabetic patients and capitalizing on the strengths of Copula functions, this article offers a fresh standpoint for comprehending and forecasting the advancement of diabetes. These findings are not only groundbreaking in theory but also possess substantial practical applicability in clinical practice.

## Data availability statement

The original contributions presented in the study are included in the article/supplementary material. Further inquiries can be directed to the corresponding author.

## Ethics statement

The National Population Health Sciences Data Center belongs to public databases. The patients involved in the database have obtained ethical approval. Users can download relevant data for free for research and publish relevant articles. Our study is based on open source data, so there are no ethical issues and other conflicts of interest.

## Author contributions

CL: Conceptualization, Data curation, Formal analysis, Methodology, Software, Validation, Visualization, Writing – original draft. HY: Conceptualization, Writing – original draft. JY: Conceptualization, Formal analysis, Investigation, Resources, Writing – review & editing. HW: Funding acquisition, Investigation, Resources, Validation, Writing – review & editing.
